# Sex and gender specific health topics in medical student learners: pulse check eight years later

**DOI:** 10.1186/s13293-021-00397-w

**Published:** 2021-10-09

**Authors:** Ann Rydberg, Matthew R. Buras, Jaxon Quillen, Virginia Miller, Juliana M. Kling

**Affiliations:** 1 Mayo Clinic Alix School of Medicine, Mayo Clinic, Scottsdale, AZ USA; 2 Department of Quantitative Health Sciences, Division of Clinical Trials & Biostatistics, Mayo Clinic, Scottsdale, AZ USA; 3 Departments of Surgery, and Physiology and Biomedical Engineering, Mayo Clinic, Rochester, MN USA; 4 Women’s Health Research Center, Mayo Clinic, Rochester, MN USA; 5 Division of Women’s Health Internal Medicine, Mayo Clinic, Scottsdale, AZ USA; 6 Center for Women’s Health, Mayo Clinic, Rochester, MN USA

## Abstract

**Background:**

An essential component of patient-centered, individualized medicine is considering how sex and gender affect mechanisms of health and disease.

**Objectives:**

To assess medical students’ current knowledge of sex and gender specific health (SGSH) concepts compared to results from the same survey in 2012 to better inform development of curricular materials for medical education.

**Methods:**

A previously designed survey tool, which assessed current knowledge of sex and gender-based medicine of medical students, was emailed to all Mayo Clinic Alix School of Medicine (MCASOM) students on Minnesota, Arizona, and Florida campuses in 2020. Descriptive and qualitative thematic results were compared to the same survey administered in 2012 to students enrolled in MCASOM. Changes in the inclusion of SGSH topics were assessed over the eight years.

**Results:**

One hundred and one of 365 (27.7% response rate) surveys were returned with 2:1 female to male respondents with representation from all 4 years. The definitions of the terms “sex” and “gender” were correctly identified by most respondents (93.1%). However, only 36% (12/33) of questions related to other medical knowledge on SGSH topics had more than a 50% correct response rate. More than half of the students reported that SGSH topics were included in Gynecology, Cardiology, Pediatrics, and Immunology. SGSH topics were reported as not being routinely covered in Neurology and Nephrology, although more students said they were in 2020 then 2012. Sixty-two percent of students favored increasing SGSH in the current curriculum.

**Conclusions:**

Medical students appear to understand the definition of and importance of SGSH in education. While some improvements in coverage by subject matter and topic area appear to have occurred as reported by medical students, opportunity remains to more fully integrate SGSH concepts in medical school curricula.

**Supplementary Information:**

The online version contains supplementary material available at 10.1186/s13293-021-00397-w.

## Introduction

Patient-centered, individualized medicine focuses on the intersection of biological factors (i.e. age, sex chromosomes, race, genetic variants, sex-specific hormones, and reproductive history), with cultural and societal factors defining gender (i.e. income, education, type of employment, life style, and environmental factors such as geographical location, temperature, altitude) that contribute to health and disease [1]. State of the art genomic studies can be used to identify individuals at risk for disease and subsequently help prevent or treat disease through a more targeted approach [2]. A fundamental component of this individualized approach is sex- and gender-based mechanisms of disease. There are innumerable data demonstrating sex differences in disease incidence, symptomology, morbidity and mortality [3]. Examples include the higher incidence of autoimmune disorders such as systemic lupus erythematous in women, the differing presenting symptomology of cardiovascular disease in women as well as the higher risk of mortality from heart attack, and the increased propensity for development of chronic obstructive pulmonary disease in female smokers [4–6]. In 2020, attention was given to sex disparities in risk, morbidity, and morality associated with the COVID-19 virus with males having greater incidence and mortality than females [7]. This difference reflects both biological differences in immunocompetency between males and females and cultural aspects associated with behaviors and co-morbidities.

The 2001 Institute of Medicine report examined the current status of the study of sex differences and recommended that sex should be considered when designing and analyzing studies in all areas [8]. The 1993 National Institutes of Health (NIH) Revitalization Act required inclusion of women in clinical studies but despite this, women are still under-represented in clinical trials and the translation of sex and gender-based science into practice remains inadequate. In 2016 the NIH announced that all grants funded by the agency must include sex as a biological variable in concept, experimental design, and evaluation [9–12].

Significant research advances have improved scientific knowledge of the interaction of sex and gender on disease and health outcomes in recent years, but these new advances are often not translated into medical education [13] 1]. When a majority of medical schools in the United States and Canada were surveyed, 70% of those who responded indicated there was not a formal integration of sex and gender specific health (SGSH) topics into their curriculum [14]. Efforts are ongoing to examine gaps in medical school curricula for sex and gender competencies and to identify strategies to embed these concepts into both curricula and clinical practice [14, 15]. Focused SGSH workshops have successfully targeted healthcare professionals of diverse disciplines and specialties; a similar approach could be used to begin integration of sex and gender-based concepts into medical professional education until more permanent integration of SGSH into curriculum is made [13, 16, 17]. Attendance at Gender Based Medicine lectures has increased awareness of and sensitivity to gender differences in medicine [18]. Medical student assessments such as USMLE Step 1 and its preparatory curriculum also contain an increasing focus on sex- and gender differences in medicine [19]. Therefore, incorporating SGSH topics into medical school will improve physician knowledge and practice of sex- and gender concepts for a more personalized approach to patient care.

As work to improve the coverage of SGSH in medical school curricula is ongoing, it is important to consider the intersectionality of biological factors with those of gender [20]. For example, while adverse childhood experiences (ACE) predispose both men and women to poorer health outcomes as adults, these poor outcomes are more severe in people of color and individuals with lower socio-economic status [21]. It is the combination of all of these factors: biology, wealth, and lived experience that impact an individual’s health. When considered in isolation neither variable provides complete information about how to enhance care. Therefore, the integration of SGSH into medical school curriculum will both augment and be enhanced by concepts of intersectionality.

A previous survey of second and fourth year Mayo medical students conducted in 2012 identified areas where SGSH topics were covered, such as gynecology, cardiology and pediatrics, but also highlighted areas were these topics were missing including nephrology, neurology and orthopedics. Students indicated they wanted these concepts embedded into existing curriculum [15]. Eight years have passed since the original survey was conducted. During this time, Mayo Clinic Alix School of Medicine (MCASOM) launched new initiatives aimed at integrating SGSH which includes longitudinal integration of the Science of Healthcare Delivery [22]. This follow-up survey of current MCASOM students offers a unique opportunity to examine if the initiatives better prepared students to address sex and gender mechanisms of disease in clinical practice.

## Methods

A duplicate of the survey administered to 2^nd^ and 4^th^ year Mayo medical students in 2012 was emailed to all current MCASOM students. The survey consisted of 34 forced-choice questions and an open-ended final question which asked for student comments or suggestions for changes in the medical curriculum related to sex and gender-based medicine. Questions addressed both practice specific guidelines and specific medical information. Four demographic questions asking students for their year in school, home campus, sex, and gender were incorporated into the survey (Additional file 1: Appendix S1).

The project was tri-site in data collection with surveys being emailed to students at the Rochester, Scottsdale, and Jacksonville sites and dual-site in its research team with team members from Rochester and Scottsdale. Students enrolled in the 2 + 2 program, the first two years occurring in either Rochester or Scottsdale and the last two in Jacksonville, were included in the results for whichever campus they were at when surveyed. Responses were received from students in Rochester and Scottsdale but none from Jacksonville. The MCASOM education committee was contacted for approval of the survey, and exempt IRB status was obtained. All communication with survey participants adhered to the policies outlined in Mayo’s Electronic Communications with Research Subjects policy.

The tool REDCap (Research Electronic Data Capture) was utilized by statisticians to administer the survey and to collect the data. The survey tool link was emailed to all 365 MCASOM students included on the tri-site student list-serv. The survey response rate was used to evaluate the success of the distribution of the assessment tool. An email reminder was sent at days 7 and 10 to maximize survey response rate. No incentive was provided, and students faced no repercussions for not completing the survey.

Fisher’s exact test was used to test the difference of distribution between sexes and other variables of interest. The statistical software R 3.6 was used for data analysis [23]. All data received by the study team from the Survey Research Center was anonymized and will remain confidential.

## Results

One hundred and one responses were received from the 365 emails sent to the full tri-site student list-serv for a response rate of 27.7%. All four years were represented with 27% of the responses being from first-year students, 26% from second-year students, 38% from third-year students, and 9% from fourth year students. The sex ratio of respondents was approximately 2:1 female to male compared to the 48.3% female to 51.7% male distribution at MCASOM. The gender ratio of respondents was also approximately 2:1 women to men (Table 1).

**Table 1:** 2020 Survey Demographic Information

Year in medical school	Student respondents
M1	27
M2	25
M3	38
M4	9
Home campus	
Rochester	38
Scottsdale	62
Jacksonville	0
Sex	
Female	63
Male	36
Prefer not to say	1
Gender	
Woman	61
Man	36
Non-Binary	1
Prefer not to say	1

Nine clinical areas were assessed to determine if topics related to sex and gender-based medicine were covered in the current curriculum (Table 2). Greater than 50% of students indicated that SGSH concepts were covered in Gynecology, Cardiology, Pediatrics, and Immunology. Forty-four percent of students indicated these concepts were addressed in Oncology, 33.7% in Gastroenterology, and 32.7% in Neurology. Less than 30% of students stated that SGSH topics were included in Nephrology and Orthopedics. Immunology, Neurology, and Nephrology, showed improvement in reported coverage over the 8 years from 2012 to the current 2020 survey. However, in Gynecology, Cardiology, and Pediatrics, medical students reported less coverage of SGSH compared to 2012.

**Table 2:** Sex and Gender in Medical Curriculum

Clinical area where sex and gender were included in curriculum	Percentage of students who replied yes		
	Present survey (%)	2012 survey aggregate results (%)	*P*-value
Gynecology	74.3	85.9	.086
Cardiology	64.4	83.1	.009
Pediatrics	51.5	77.5	< .001
Immunology	50.5	45.1	.537
Oncology	43.6	60.6	.031
Gastroenterology	33.7	49.1	.042
Neurology	32.7	29.6	.740
Nephrology	29.7	25.4	.606
Orthopedics	27.7	31.0	.733

### Medical knowledge and practice guidelines

**Fig. 1:**
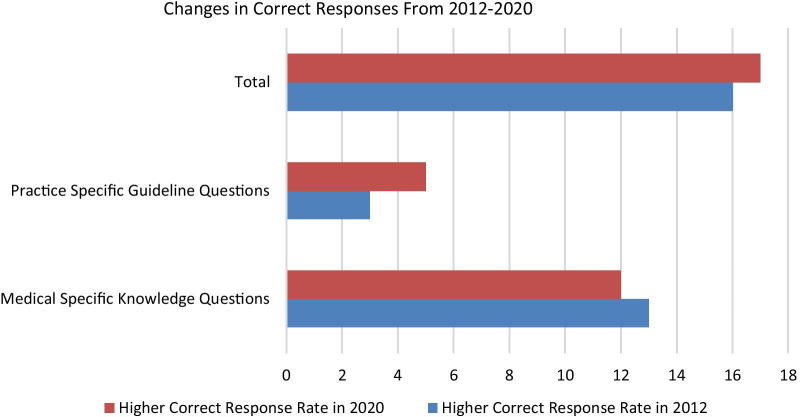
Changes in Correct Response from 2012 to 2020

Within the survey questions assessing medical knowledge and familiarity with practice guidelines, seventeen of the thirty-three items had more correct responses in the current survey than in 2012 (Fig. 1). Sixteen questions showed higher correct responses in the previous survey. Of 2020 respondents, 93.1% identified that the terms sex and gender should be differentiated when discussing the biological basis of disease (p-value: 0.775). In both surveys most students identified that, in general, current prevention and treatment management strategies do not take into consideration biological differences between men and women (81.2%), and that the Cochrane Database does not have sufficient evidence about treatment outcomes for women as for men (94.0%). Examples of practice specific guideline related questions are shown in Table 3, and examples of medical knowledge questions are shown in Table 4.

**Table 3:** Practice Specific Guideline Question Examples

Sex and gender-specific items related to practice guidelines (correct answers are noted)	Percentage of students selecting each answer			
	Present survey	2012 Survey (4th year students; 2nd year students)	2012 aggregate survey results (%)	*P*-value
Practice guidelines are often developed based on results of clinical trials. Analyzing clinical studies by sex can include: (select multiple) (all are correct)				
a. Reporting the sex of study subjects	83.2%	34.4%; 26.3%	30.0	< .001
b. Incorporating sex in multivariant analyses	92.1%	50.0%; 36.8%	42.9	
c. Analyzing results by sex	94.1%	75.0%; 47.4%	60.0	
d. Reporting null findings	69.3%	21.9%; 0.0%	10.0	
Differences in fat distribution between men and women affect circulating concentrations of pharmacological therapy				.440
TRUE (correct)	94.1%	84.8%; 94.6%	90.0	
FALSE	5.9%	15.2%; 2.7%	8.6	
Don't Know	Not included	0%; 2.7%	1.4	
In general, current prevention and treatment management strategies take into consideration biological differences between men and women				.111
TRUE	18.8%	25.8%; 31.6%	29.0	
FALSE (correct)	81.2%	74.2%; 65.8%	69.6	
Don't Know	Not included	0%; 2.6%	1.4	
The Cochrane Database has as much evidence about treatment outcomes for women as for men				.162
TRUE	6.0%	7.1%;10.56%	9.1	
FALSE (correct)	94.0%	92.9%; 84.2%	87.9	
Don't Know	Not included	0%; 5.3%	3.0

**Table 4:** Medical Specific Knowledge Question Examples

Examples of items related to medical specific information (correct answers are noted)	Percentage of students selecting each answer			
	Present survey (%)	2012 Survey (4th year students; 2nd year students)	2012 aggregate survey results (%)	*P*-value
More men than women die of cardiovascular disease in the United States each year				.283
a. Disagree (correct)	23.8	34.4%; 11.1%	22.1	
b. Agree	39.6	50.0%; 63.9%	57.4	
c. Not Sure	36.6	15.6%; 25.0%	20.6	
Idiopathic pulmonary hypertension is a rare but fatal disorder the occurrence of which shows				.001
b. Greater prevalence in women than men (correct)	60.0	84.4%; 81.6%	82.9	
a. No sex difference	3.0	6.2%; 2.6%	4.3	
c. Don’t know	37.0	9.4%; 15.8%	12.9	
Women respond to the flu vaccine by developing higher titers of antibodies than men				.545
b. Agree (correct)	13.9	12.9%; 5.1%	8.6	
a. Disagree	14.9	16.1%; 12.8%	14.3	
c. Not Sure	71.3	71.0%; 82.1%	77.1	

In the open-response question 66 students provided additional responses with suggestions for changes in the medical curriculum related to SGHS. Sixty-three of those responses were in favor of increased SGSH. Examples of the feedback provided are shown in Table 5.

**Table 5:** Student Feedback on Sex and Gender Specific Health

Examples of feedback on coverage sex and gender-based topics
Current knowledge
I did not realize there were so many differences between the sexes until taking this survey. I definitely think we should be taught this throughout our medical education
I pretty much had no idea what the answer was to any of these. That said, I haven’t even taken many of these courses and didn’t know what most of these conditions were or if a professor would have stressed sex differences in the manifestation or management of those conditions
Many of these questions I had to make an educated guess on—would be better if more of these differences (or lack thereof) were actively taught in medical school
Suggestions for improvement
All lectures should include differences in presentation, management and diagnosis between men and women; it should be part of the basic lecture when learning about any disease in the first two years of medical school
More OSCE (Objective Structured Clinical Experience) practice would be very helpful
Please make it as longitudinal as possible
We need more scientific information about how diseases/drugs/interventions differ between xx and xy patients

## Discussion

It is clear that sex- and gender differences exist and influence patient outcomes, and, thus, must be taught in medical school. Incorporating SGSH as a crucial part of personalized medicine will improve patient outcomes in a variety of disease states including cardiovascular disease, malignancy, and neurodegenerative diseases [24]. Even a single training session can improve understanding and application of SGSH topics as verified by pre-and post-assessments [16]. Indeed, one of the written comments to the survey indicated that the respondent didn’t have knowledge of sex and gender differences until taking the survey.

SGSH topics have not been routinely incorporated into medical curricula. In comparing student reported SGSH content from 2012 to 2020 in the curriculum and in spite of efforts to increase course content of the related topics, the coverage of SGSH topics showed variability with the majority of subject areas (6/9) reported to have less coverage than in 2012 while only coverage of Neurology, Nephrology, and Immunology were reported to have increased. However, when comparing the number of correct responses to medical knowledge and practice guideline questions, 17/33 questions showed more correct responses in 2020 (Fig. 1). Therefore, there appears to be an increase in students understanding of SGSH concepts, and students are learning about SGSH concepts even when it is not directly acknowledged. Important medical knowledge questions such as the risks pre-eclampsia poses to mothers later in life or the sex-specificity of irritable bowel syndrome (IBS) were well answered by the students. The discrepancy between student reporting and correct answers to knowledge-based questions emphasizes the importance of being intentional with inclusion of SGSH educational materials. The fact that students correctly respond to some questions despite indicating that these topics were not well taught could indicate that interested students are reading material on their own or gaining the information from sources other than structured curriculum. Although the improvements in the results of the survey over the 8 years are encouraging, they provide the impetus for improvement in clarity of instruction and perhaps the need to address alternative educational approaches to the subject matter.

In 2020 as well as 2012, students had high rates of correct responses to questions relating to practice specific guidelines. The questions related to practice guidelines had a greater than 60% correct response rate, and all showed increased correct responses in 2020 compared to 2012. As further research demonstrates clear sex and gender differences in disease, it would be expected that students’ exposure to these topics in their curriculum would rise, as well as their ability to correctly answer questions regarding this content.

The questions that most students answered incorrectly provide insight into areas where SGSH content could be improved. For example, given that 70.3% of students attested that SGSH topics were not included in Nephrology, it is not surprising that less than half of students correctly identified that progressive loss of kidney function occurs faster in men. Likewise, students struggled with the true/false questions asking whether gastric secretion is higher in men than women (44% answered incorrectly), which is further supported by the fact that only 33.7% of students said that Gastroenterology addressed SGSH concepts.

There are many resources available to streamline the integration of SGSH concepts into medical student curriculum and several examples of successful integration carried out both in medical school and later levels of training. Medical textbooks such as *How Sex and Gender Impact Clinical Practice, Sex- and Gender-Based Women’s Health,* and *Principles of Gender-Specific Medicine* provide accessible and relevant information for a variety of clinical topics [25–27]. Modules are available online from many sites including the NIH Office of Research on Women's Health (ORWH), the Laura Bush Institute’s Sex and Gender-specific Health Curriculum, and U.S. Food and Drug Administration (FDA) webinars [13, 22]. Tools such as these will allow for easy supplementation of existing curricula with SGSH concepts. One example of successful integration of SGSH curricular integration is the elective-course “Sex- and Gender-Based Medicine: an Overview,” offered by Alpert Medical School [13]. The popular course consisted of a hybrid learning model in which students attended lectures given by experts in SGSH, and supplemented their learning with the tools discussed above [13]. Ideally, SGSH topics will be incorporated throughout the curricula; however, these tools can be useful to begin the process of integration.

Importantly, the degree of student interest in improving coverage of SGSH should be noted. In fact, over sixty percent of the students chose to write in an additional response asking for more coverage of these topics. This information is incredibly important as student-driven change has the potential to transform curricula. For example, the University of Illinois College of Medicine-Chicago (UICOM-Chicago) has recently instituted a new Student Curricular Board that has been very successful and well-received in improving and diversifying the curriculum [28]. Involving all stakeholders, students and educators alike, in curricular change will lead to more effective and well-received change [22].

Improving the integration of SGSH concepts into medical student education and subsequently clinical practice is essential to provide evidence based and personalized patient care. Educators must first be educated about the importance of SGSH and informed about the status of integration of these concepts into current curriculum before progress can be made. The results of this survey should by no means reflect badly on the medical education curriculum at MCASOM or its students. MCASOM is ranked as a top medical schools and is one of the most selective medical schools in the US. Efforts have been made through the introduction of the Science of Healthcare Delivery curriculum to expand student’s knowledge of socioeconomic, racial, and gender-expression based disparities in care [29]. Therefore, we mean this current study to reflect the current trends in sex- and gender-based medicine in medical student education more broadly.

### Strengths and limitations

This study longitudinally evaluated curriculum in the area of SGSH using the same survey over a period of eight years. Furthermore, it included representation from three geographically diverse student populations under the umbrella of a single medical school. Because the study compared the results of the same survey applied in both 2012 and 2020, it was not possible to correct any ambiguity in the survey questions. The inclusion of Gynecology is important as this specialty was traditionally considered the only one of sex-based interest. With the emphasis on gender and issues related to LBGTQ + sexuality care, the specialty takes on a gender-based components such as education, culture, ad socioecomomic status. Although the survey contained more emphasis on sex differences than gender differences such that the intersectionality of biological factors with those of gender including concepts of LGBTQ + status, ethnicity, and socio-economic status will provide a more holistic, patient-centered approach to SGSH. Likewise, including patient sex and gender into studies assessing the effects of race, age, socio-economic status, etc. helps to obtain more nuanced results [1]. A recent work by Jaehn et al. argues for the importance of considering intersectionality when determining study representativeness and identifies how crucial it will be for future research to use an intersectional approach to draw accurate conclusions about study cohorts to improve future practice guidelines [30].

A limitation of the study is the response rate. The original survey was delivered in person to 1^st^ and 2^nd^ year students at the end of the school year term. However, the current survey was delivered electronically which perhaps contributed to the lower response rate. While, the current survey consisted of more responses (101) than the 2012 survey, it was given to all years of medical school and some topics may have yet to have been covered for individual student programs (77). As this study only surveyed students at a single medical school program, it is difficult to generalize results to other programs. It is also limited to medical student perception and recall and findings are not corroborated by curriculum review.

Most of the respondents were female and women and female medical learners are more likely to consider gender and sex in providing care [31]. A large nationwide survey found that male medical students were more likely to state that they had received more SGSH education than female medical students who had received the same education [32]. This discrepancy may indicate that female students would be more likely to request more training than their male counterparts which could influence the results of our study, particularly the student interest in increasing SGSH coverage.

Because this study is limited by the small sample size given remote delivery of the study at a single medical school, the p-values comparing the two surveys are variable making statistically significant conclusions difficult. However, the study does provide a snapshot view of the current state of SGSH at this medical school upon which informed recommendations can be made to improve the curriculum.

## Perspectives and significance

Concepts of SGSH affect treatment decisions and health outcomes of almost every area of medicine. Not only are these concepts important for clinical practice but as sex and gender research and knowledge expands, these concepts are being included on licensing and board examinations. This study shows that despite advancements in the integration of SGSH topics into curriculum, there are still many opportunities to enhance student understanding of these important concepts. Notably, students are interested and motivated to learn more SGSH concepts to optimize their ability to provide specific, tailored care to future patients. Successful incorporation of SGSH into educational programs in medical schools will allow graduating students to practice high-quality, evidence-based medicine.

## Supplementary Information

**Additional file 1.** Sex and Gender in the Medical Curriculum Beta Test, MCASOM students, 2020.

## Data Availability

All data generated or analyzed during this study are included in this published article and its supplementary information file.
