# Supplementary material for: Sex and gender specific health topics in medical student learners: pulse check eight years later
Source: Biol Sex Differ. 2021 Oct 9;12:53. doi: 10.1186/s13293-021-00397-w (PMC8501372; doi:10.1186/s13293-021-00397-w)
Supplement: Supplementary file 1 — Additional file 1. Sex and Gender in the Medical Curriculum Beta Test, MCASOM students, 2020. [file 13293_2021_397_MOESM1_ESM.docx]

**Additional file 1: Appendix S1**

Sex and Gender in the Medical Curriculum

Beta Test, MCASOM Students, 2020

**Section 1**

**Select one or more correct answers for each of the following questions:**

#### 1. Select any of the following field(s) where biological sex differences which apply to the accurate diagnosis and/or treatment of disease have been included in your medical curriculum:

a. Cardiology

b. Oncology

c. Pediatrics

d. Orthopedics

e. Gynecology

f. Neurology

g. Nephrology

h. Immunology

i. Gastroenterology

#### 2. The terms sex and gender …

a. Can be used interchangeably when discussing the biological basis of disease

b. Should be distinguished when discussing the biological basis of disease

c. Can be used interchangeably when discussing both social and biological aspects of disease

d. Don’t know

#### 3. Practice guidelines are often developed based on results of clinical trials. Analyzing clinical studies by sex can include …

a. Reporting the sex of study subjects

b. Incorporating sex in multivariate analyses

c. Analyzing results by sex

d. Reporting null findings

#### 4. Women with angina symptoms often go untreated because: …

a. Women may suffer from atypical symptoms such as nausea, dizziness and fatigue

b. Physicians are often confused by atypical early symptoms and tend to overlook them

c. More men than women die of cardiovascular diseases

d. Not sure

**Section 2**

**Select the best answer for each question below unless directed to select multiple answers:**

#### 5. What are Phase III clinical trials?

a. Randomized controlled trials of 20 to 100 patients to test drug safety.

b. The first stage of testing drugs on small groups of healthy volunteers.

c. Multicenter trials on large patient groups.

d. I don’t know.

#### 6. More men than women die of cardiovascular disease in the U.S. each year.

a. Disagree

b. Not sure

c. Agree

#### 7. Most cancer-related deaths in men worldwide are caused by

a. Breast cancer

b. Prostate cancer

c. Lung cancer

d. Leukemia

e. Skin cancer

#### 8. Most cancer-related deaths in women in the US are caused by

a. Breast cancer

b. Prostate cancer

c. Lung cancer

d. Leukemia

e. Skin cancer

#### 9. Select all cancers that men do not get ():

a. Prostate cancer

b. Lung cancer

c. Cervix cancer

d. Breast cancer

e. Colorectal cancer

#### 10. In the development of heart failure….

a. Women tend to have concentric myocardial hypertrophy with systolic dysfunction.

b. Women tend to have eccentric myocardial hypertrophy with diastolic dysfunction.

c. Women tend to have concentric myocardial hypertrophy with rather preserved systolic function.

d. Men tend to have concentric myocardial hypertrophy with rather preserved systolic function.

e. Haven’t a clue

#### 11. In medical complications of alcoholism:

a. Women display more severe cognitive and motor impairment with lower alcohol exposure compared with men.

b. Cirrhosis develops after fewer years of heavy drinking in men than women.

c. Alcohol-induced cardiomyopathy develops after fewer years of heavy drinking in men than women.

d. Alcohol-induced peripheral neuropathy develops after fewer years of heavy drinking in women than men.

e. Not sure

#### 12. Progressive loss of kidney function occurs faster in

a. Men.

b. Both sexes progress at equal rate.

d. Men progress in general faster, although it depends on the background etiology.

e. Women progress in general faster, although it depends on the background etiology.

#### 13. Chronic clinical pain is …

a. More common in men.

b. As common in men as in women.

c. More common in women.

#### 14. Sex chromosomes – particularly the Y chromosome – appear to be tied to predisposition for:

a. Autism.

b. Hypertension.

c. Renal disease.

#### 15. Women respond to the flu vaccine by developing higher titers of antibodies than men.

a. Disagree

b. Not sure

c. Agree

#### 16. Pre-eclampsia of pregnancy predisposes:

a. The baby to cardiovascular disease in adulthood.

b. The mother to cardiovascular disease later in life.

c. Male babies more than female babies to greater cardiovascular risk at birth.

d. no future health risk once resolved.

#### 17. Hepatitis B and C are very common infectious diseases worldwide. Chronic infections can lead to liver diseases, liver failure or hepatocellular carcinomas. Who is at greater risk and why?

a. Men, since estrogen appears to act as a protective factor

b. Men, since testosterone appears to act as a risk factor

c. Women, since testosterone appears to act as a protective factor

d. Not sure

#### 18. Idiopathic pulmonary hypertension is a rare but fatal disorder the occurrence of which shows

a. No sex difference.

b. Greater prevalence in women than men.

c. Don’t know

#### 19. Lower esophageal cancer affects both sexes but is

a. More prevalent in males.

b. More prevalent in females.

c. equally prevalent in males and females

d. Don’t know

#### 20. Hepatitis B (HBV) is transmitted by blood and bodily fluids. Several factors influence the risk of developing chronic disease. Who is more at risk to develop a ***chronic*** HBV infection?

a. A 40 year old women who contracted HBV by sexual intercourse.

b. A 35 year old male drug user who contracted HBV by use of intravenous drugs.

c. A 6 month old girl who contracted HBV due to the use of non-sterilized medical equipment.

d. Don’t know

#### 21. It has been proposed that irritable bowel syndrome (IBS) maintains a sex specificity based on which of the following factors?

a. Women tend to experience pain in the abdominal region in a different manner than men subjected to the same stimulus.

b. Women tend to perceive a disease affecting their bowel movements with more embarrassment and less confidence than men.

c. Women tend to somatize stressors in a different manner than men and this affects modulation of gastrointestinal functions by the autonomic nervous system.

d. All of the above

e. None of the above

#### 22. Hepatic enzymes play a role in physiologic digestive processes and in detoxification. Which of the following statement(s) is true?

a. The activity of cytochromes in the liver differs between women and men.

b. Women produce less efficient liver enzymes than men.

c. Cytochromes in men are more active than in women.

d. All of the above

e. None of the above

#### 23. Which of the following statements about sex differences at start of dialysis therapy is **FALSE**?

a. More men than women start dialysis therapy every year.

b. Men die more often than women due to complications with dialysis.

c. Men on dialysis usually have a worse risk profile for renal disease than women.

d Men on dialysis are more likely to experience an adverse cardiovascular event.

#### 24. Which statement concerning the incidence of cardiovascular disease (CVD) is **FALSE**.

a. Under the age of 50, the incidence of CVD and risk of death are higher in men than in women.

b. In women over 50, there is a rapid increase in the incidence of CVD.

c. At age 65 the incidence of CVD is the same for both men and women.

d. Over the past decade, death from CVD in both men and women has increased in all age categories over 55 years.

#### 25. Which statement concerning “heart failure” (HF) is **FALSE**?

a. Echocardiographically measured “heart failure with preserved ejection fraction” (HFpEF) is more frequent in women than in men.

b. Survival in systolic HF is better in women – independent of therapy.

c. Cardiomyopathies are a rare but severe cause of HF leading to 50% of all transplantations for end stage heart failure in both men and women.

d. Myocardial infarction is a greater risk factor for heart failure in women compared to men.

**Section 3**

**True or False**

#### 26. Multiple sclerosis is as common in men as in women, but men have a worse prognosis.

#### 27. Depression is more common in women, but treatment is more successful for men.

#### 28. Alcoholism is more common in men, but treatment is more successful for women.

#### 29. Gastric secretion is higher in men than women.

#### 30. Differences in fat distribution between men and women affect circulating concentrations of pharmacological therapy.

#### 31. Eight of the last 10 drugs withdrawn from the market in the US had more side effects in men.

#### 32. In general, current prevention/treatment management strategies take into consideration biological differences between men and women.

33. All drugs are equally effective whether given at the luteal or follicular phase of the menstrual cycle.

34. The Cochrane Data Base has as much evidence about treatment outcomes for women as for men.

**Please provide any comments or suggestions for changes in the medical curriculum related to sex/gender-based medicine:**

**Demographics**

1. What year are you in medical school?

a. M1

b. M2

c. M3

d. M4

2. Where is your home campus?

a. Rochester

b. Arizona

3. What is your sex?

a. Female

b. Male

c. Intersex

d. Prefer not to say

4. What is your gender?

a. Woman

b. Man

c. Transgender

d. Non binary

e. Other

f. Prefer not to say
